# Bioprospecting of *Ocotea minarum *(Laurales: Lauraceae) by ethanolic extract in control of strains of gender *Candida*

**DOI:** 10.1186/1753-6561-8-S4-P21

**Published:** 2014-10-01

**Authors:** Allan Belarmino Rodrigues, Bruna de Paula Bicudo, Laura Wiebusch, Rodrigo Raghiant Borges, Adriana Araújo de Almeida, Kelly Mari Pires de Oliveira

**Affiliations:** 1 Faculty of Biological and Environmental Sciences (FCBA), Federal University of Dourados Region (UFGD), Dourados, Brazil; 2 Faculty of Health Science (FCS), Federal University of Dourados Region (UFGD), Dourados, Brazil; 3 Faculty of Biological and Environmental Science (FCBA), Federal University of Dourados Region (UFGD) - Unit II: Highway Dourados - Itahum, Km 12; 79.804-970; Dourados - MS, Brazil

## Background

The *Ocotea minarum *is a plant native to Cerrado and found in abundance in this Brazilian biome. Belonging to the family Lauraceae is popularly known in the region as "Shin-broom". It is a medium-sized tree, occurring in several states, among them, the Mato Grosso do Sul. Due to the presence of some compounds such as tannins, steroids, triterpenes and flavonoids in its shell, it may have antifungal activity [1]. There are records of the popular use of its bark in the form of infusion and aqueous extract for candidiasis treatment. However, few studies relating to biological effects such compounds indicated [2]. Candidiasis is an opportunistic infection caused by Candida species, being the most common agent is *Candida albicans*. The disease can affect your mouth, eyes and vaginal mucosa. Vaginal candidiasis affects a high proportion of women in adulthood, it is estimated that approximately 75% of these have at least one episode of fungal vulvovaginitis in your life [3]. The aim of this study was to determine whether the ethanol extract of *Ocotea minarum *shows antifungal activity against *Candida spp*.

## Methods

The shell of the *Ocotea minarum *dried and pulverized was mixed in 90 mL of 95% ethanol and left at 25 ° C for 72 h. The plant extract filtrate was completely evaporated at 35 º C and lyophilized. The extract was suspended in dimethyl sulfoxide to 2048 mg / mL final concentration, and the initial concentration was 4 mg / mL, was then added in RPMI 1640 medium and poured into 100 mL of microdilution plate with 96 wells. Immediately after it was added 100 mL of inoculum at a concentration of 0.5 McFarland (108 CFU / ml) in the wells. The microbial suspension was used as positive control, while broth containing the extract as a negative control was used, MIC values were analyzed with the lowest concentration of the extract in the wells of microdilution plate showed no turgor after inoculation. Samples were removed from each well of the plate microdilution MIC and perforated in a petri dish containing Sabouraud Dextrose agar (Difco) for evaluating the minimum fungicidal concentration (MFC) [4].

## Results and conclusions

Based on the criteria of Araújo [5], the evaluation of the antifungal activity of ethanol extract of *Ocotea minarum*, observed effective antifungal activity against the strains of *Candida tropicalis *in a concentration of 64 mg / mL and *Candida krusei *concentration of 1024 mg / ml. Strains of *Candida albicans *and *Candida glabrata *were not growth inhibited by the extract. With these results we can conclude that the ethanol extract of the bark of *Ocotea minarum *studied shows antifungal activity and therapeutic potential feasible and cost effective. The results were obtained by in vitro studies, aiming later conducting research in vivo, so that this extract can be used to obtain a bioactive principle in the production of pharmaceuticals and cosmetics, aimed at curing diseases clinics related to the genus *Candida*.

## References

[B1] Silva MRO Antifungal activity detection of mangrove plants extracts from Vila Velha, Itamaracá-PE Itamaracá-PE 2004 39p

[B2] Silva KL Filho VC Plants of gender Bauhinia - Chemimcal Composition and pharmacological potential Quim Nova 2002 25 3 449 454 10.1590/S0100-40422002000300018

[B3] Ferraza MH Maluf MLF Consolaro MEL Shinobu CS Svidzinski TIE Batista MR Characterization of yeasts isolated from the vagina and their association with vulvovaginal candidiasis in two cities in southern Brazil Rev Brasi Ginec Obst 2005 27 58 63 10.1590/S0100-72032005000200003

[B4] Clinical and Laboratory Standards Institute (CLSI) Reference method for broth dilution antifungal susceptibility testing of yeasts: approved standard M27-A3 2008 3 Wayne: Clinical and Laboratory Standards Institute

[B5] Araújo MGF Chemical constituents of the methanolic extract of leaves of Leiothrix spiralis Ruhland and their antimicrobial activity Molecules 2011 16 10479 10490 10.3390/molecules161210479 22179427 PMC6264751

